# Correlation between serologic parameters and disease activity of IgG4-related disease: Differences between patients with normal and elevated serum IgG4 concentrations

**DOI:** 10.3389/fimmu.2022.1020459

**Published:** 2022-10-12

**Authors:** Oh Chan Kwon, Min-Chan Park, Yong-Gil Kim

**Affiliations:** ^1^ Division of Rheumatology, Department of Internal Medicine, Yonsei University College of Medicine, Seoul, South Korea; ^2^ Division of Rheumatology, Department of Internal Medicine, University of Ulsan, College of Medicine, Asan Medical Center, Seoul, South Korea; ^3^ Convergence Medicine Research Center, Asan Institution for Life Science, Asan Medical Center, Seoul, South Korea

**Keywords:** IgG4-related disease, disease activity, IgG4, serology, correlation

## Abstract

**Objective:**

We aimed to identify serologic parameters that correlate with the disease activity of IgG4-related disease (IgG4-RD) in patients with normal and elevated serum IgG4 concentrations, respectively.

**Methods:**

This retrospective cohort study included 148 patients with IgG4-RD. Patients were categorized into normal (≤201 mg/dL) and elevated (>201 mg/dL) serum IgG4 concentration groups. Disease activity was assessed using the IgG4-RD responder index (RI). The correlations between IgG4-RD RI and serologic parameters (erythrocyte sedimentation rate [ESR], C-reactive protein, C3, C4, IgG4 concentration, IgG concentration, and IgG4/IgG ratio) were evaluated in each group, using Spearman’s correlation coefficient.

**Results:**

Of the 148 patients with IgG4-RD, 38 (25.7%) and 110 (74.3%) patients were categorized into the normal and elevated serum IgG4 concentration groups, respectively. In the normal serum IgG4 concentration group, IgG concentration was the only serologic parameter that showed a significant correlation with IgG4-RD RI (rho=0.411, p=0.013). However, in the elevated serum IgG4 concentration group, ESR (rho=0.196, p=0.041), C3 (rho=-0.432, p<0.001), C4 (rho=-0.363, p=0.001), IgG4 concentration (rho=0.423, p<0.001), IgG concentration (rho=0.224, p=0.020), and IgG4/IgG ratio (rho=0.328, p=0.001) correlated with IgG4-RD RI. The combination of C3 and IgG4 concentration (rho=0.509, p<0.001) had the strongest correlation with IgG4-RD RI in this group.

**Conclusion:**

Among the serologic parameters tested, IgG concentration was the only parameter that correlated with IgG4-RD RI in patients with normal serum IgG4 concentrations, whereas multiple parameters correlated with IgG4-RD RI in those with elevated serum IgG4 concentrations. The combination of C3 and IgG4 concentration had the strongest correlation coefficient in the latter group.

## Introduction

Immunoglobulin G4-related disease (IgG4-RD) is an immune-mediated condition characterized by fibroinflammatory lesions that can affect virtually any organ (1–3). Although elevation of serum IgG4 concentrations was once considered a prerequisite for the diagnosis of IgG4-RD, it is now known that elevated serum IgG4 concentration is neither sufficiently sensitive nor specific for the diagnosis of IgG4-RD (2, 4). A substantial proportion of patients with IgG4-RD have normal serum IgG4 concentrations (4–6); in a cohort consisting of 125 patients with IgG4-RD, 48.5% of patients had normal serum IgG4 concentrations, even among patients with clinically active disease (6).

IgG4-RD responder index (RI) is a validated disease activity assessment tool widely used for IgG4-RD (7). The scoring system consists of 26 domains (24 standard organs, constitutional symptoms, and other organs) that are scored separately and then summed to calculate the IgG4-RD RI (7). Although reliable and valid as a disease activity assessment tool (7), it is difficult to accurately score the IgG4-RD RI if laboratory tests and imaging studies to assess organ involvement status are not fully completed. Full laboratory and imaging assessments at every visit are not feasible in routine clinical practice. Hence, identifying a simple serologic biomarker that is readily available in routine clinical practice and correlates well with the disease activity of IgG4-RD could be helpful. Serum IgG4 concentration has been traditionally suggested as a biomarker for the disease activity of IgG4-RD (8). However, data on the use of serum IgG4 concentration as a biomarker for disease activity of IgG4-RD has shown mixed results (9–11). Therefore, the validity of serum IgG4 concentration as a biomarker of disease activity, when universally applied to patients with IgG4-RD as a whole, is controversial. It is particularly uncertain whether serum IgG4 concentration could be used as a biomarker for disease activity of IgG4-RD in a subset of patients with normal serum IgG4 concentrations. Data on whether serum IgG4 concentration correlates stronger or weaker with disease activity of IgG4-RD in particular subsets of patients are lacking; to better assess this, it is necessary to analyze patients with normal serum IgG4 concentrations and those with elevated serum IgG4 concentrations separately.

In this study, using a cohort of patients with IgG4-RD, we categorized patients into those with normal serum IgG4 concentrations and those with elevated serum IgG4 concentrations and assessed the correlation between serum IgG4 concentration and disease activity in each group. We also tested other easily accessible serologic parameters, including erythrocyte sedimentation rate (ESR), C-reactive protein (CRP), complement 3 (C3), complement 4 (C4), IgG concentration, and IgG4/IgG ratio, as potential biomarkers for disease activity in each group.

## Methods

### Study population

Patients who were newly diagnosed with IgG4-RD between 2011 and 2021 at two referral hospitals were retrospectively included for analysis. All included patients fulfilled the 2019 American College of Rheumatology (ACR)/European League Against Rheumatism (EULAR) classification criteria for IgG4-RD (12). Data on the following variables at the time of IgG4-RD diagnosis were reviewed: age, sex, involved organs, ESR, CRP, C3, C4, serum IgG4 concentration, serum IgG concentration, and serum IgG4/IgG ratio. Serum IgG4 concentrations were measured by nephelometry using Siemens assay (Siemens Healthcare Diagnostics, Malburg, Germany). The manufacturer’s reference range of serum IgG4 concentration was 3–201 mg/dL. Patients were categorized into the normal serum IgG4 concentration (≤ 201 mg/dL) and elevated serum IgG4 concentration (> 201 mg/dL) groups according to their serum IgG4 concentrations. This study was approved by the Institutional Review Board (IRB) of Gangnam Severance Hospital (IRB No: 3-2022-0247). Owing to the retrospective design of the study, the requirement for informed consent was waived.

### Disease activity

We used the IgG4-RD responder index (RI) as the gold standard of disease activity assessment (7). All patients had undergone laboratory and imaging studies for the diagnostic workup of IgG4-RD, and the scoring of each domain of IgG4-RD RI was assessed comprehensively based on the clinical, laboratory, and imaging findings at the time of diagnosis. Although the serum IgG4 concentration was included in the earlier version of IgG4-RD RI (13), this domain was removed in the latest version (7). As we used the latest version of IgG4-RD RI, serum IgG4 concentration was not included in the scoring of IgG4-RD RI.

### Statistical analysis

Continuous variables following normal or non-normal distribution are expressed as mean (± standard deviation [SD]) or median (interquartile range [IQR]), respectively, and categorical variables are expressed as numbers with percentages. For comparison between the two groups (normal serum IgG4 concentration vs. elevated serum IgG4 concentration), we used the independent two-sample t-test or Mann–Whitney U test for continuous variables following normal or non-normal distribution, respectively. For comparison of categorical variables, the χ2 test or Fisher’s exact test was used. Correlations between IgG4-RD RI and serologic parameters (ESR, CRP, C3, C4, IgG4 concentration, IgG concentration, and IgG4/IgG ratio) were evaluated using the Spearman’s correlation coefficient. Correlations were assessed in the normal serum IgG4 concentration and elevated serum IgG4 concentration groups. Multivariable linear regression analysis with backward elimination was conducted to select a combination of serologic parameters that could potentially have a stronger correlation with IgG4-RD RI. All variables that significantly correlated with IgG4-RD RI in the correlation analysis were included in the multivariable model, except for the serum IgG4/IgG ratio, which was excluded due to multicollinearity with serum IgG4 concentration and serum IgG concentration. The variables selected in the multivariable analysis were combined by multiplying each variable by its respective β coefficient, and then summing them. A p-value of < 0.05 was considered statistically significant. All analyses were conducted using SPSS software (version 25.0; IBM Corp., Armonk, NY, USA). Figures were generated using GraphPad Prism (version 7.0; GraphPad Software Inc., San Diego, CA, USA).

## Results

### Baseline characteristics of the patients

A total of 148 patients with IgG4-RD who fulfilled the 2019 ACR/EULAR classification criteria for IgG4-RD were included for analysis. The median value of the total points of the 2019 ACR/EULAR classification criteria for IgG4-RD was 26.0 (23.0–34.0). The baseline characteristics of the patients are shown in **Table 1**. The mean age of the patients was 57.3 ( ± 11.5) years, and 68.2% were male. The organ most commonly involved was the lymph nodes (36.5%), followed by orbits and lacrimal glands (31.8%), salivary glands (29.1%), kidney (16.2%), retroperitoneum (15.5%), pancreas (13.5%), and lung (13.5%). According to the number of organs involved, 48.0%, 23.0%, and 29.1% of the patients had one, two, and three or more organs involved, respectively. The median (or mean) values of the serologic parameters were as follows: ESR, 34.0 (15.0–61.0) mm/h; CRP, 2.4 (0.5–10.4) mg/L; C3, 102.3 ( ± 31.9) mg/dL; C4, 23.5 ( ± 11.0) mg/dL; IgG4 concentration, 439.0 (179.3–1157.5) mg/dL; IgG concentration, 1535.9 (1251.8–1975.0) mg/dL; and IgG4/IgG ratio, 0.28 (0.13–0.61). According to the serum IgG4 concentrations, 38 (25.7%) and 110 (74.3%) patients were categorized into the normal and elevated serum IgG4 concentration groups, respectively. The median value of IgG4-RD RI was 5.0 (4.0–8.0).

**Table 1:** Characteristics of the 148 patients diagnosed with IgG4-related disease.

	N = 148
Age, years, mean ( ± SD)	57.3 ( ± 11.5)
Male sex, n (%)	101 (68.2)
Organ involvement, n (%)	
Lymph nodes	54 (36.5)
Orbits and lacrimal glands	47 (31.8)
Salivary glands	43 (29.1)
Kidney	24 (16.2)
Retroperitoneal fibrosis	23 (15.5)
Pancreas	20 (13.5)
Lung	20 (13.5)
Aorta	12 (8.1)
Bile duct	8 (5.4)
Prostate	6 (4.1)
Sinusitis	6 (4.1)
Pituitary gland	4 (2.7)
Others*	25 (16.9)
Number of organs involved, n (%)	
1 organ involved	71 (48.0)
2 organs involved	34 (23.0)
≥3 organs involved	43 (29.1)
ESR, mm/h, median (IQR)	34.0 (15.0–61.0)
CRP, mg/L, median (IQR)	2.4 (0.5–10.4)
C3, mg/dL, mean ( ± SD)	102.3 ( ± 31.9)
C4, mg/dL, mean ( ± SD)	23.5 ( ± 11.0)
IgG4 concentration, mg/dL, median (IQR)	439.0 (179.3–1157.5)
Normal serum IgG4 concentration, n (%)	38 (25.7)
Elevated serum IgG4 concentration, n (%)	110 (74.3)
IgG concentration, mg/dL, median (IQR)	1535.9 (1251.8–1975.0)
IgG4/IgG ratio, median (IQR)	0.28 (0.13–0.61)
IgG4-RD RI, median (IQR)	5.0 (4.0–8.0)

ESR, erythrocyte sedimentation rate; CRP, C-reactive protein; IgG4-RD, IgG4-related disease; RI, responder index.

*Detailed numbers of patients with other organ involvement are as follows: pleura (n = 4), heart (n = 3), mesentery (n = 3), paraspinal mass (n = 3), pericardium (n = 3), testicle (n = 3), gallbladder (n = 2), pharynx (n = 2), adnexa (n = 1), bladder (n = 1), colon (n = 1), liver (n = 1), nasal cavity (n = 1), peritoneum (n = 1), skin (n = 1), and stomach (n = 1).

### Comparison between patients with normal and elevated serum IgG4 concentrations

Compared with patients with normal serum IgG4 concentrations, those with elevated serum IgG4 concentrations were older (52.5 [ ± 13.4] years vs. 58.9 [ ± 10.3] years, p = 0.009), more commonly had salivary gland (7.9% vs. 36.4%, p = 0.001), pancreas (2.6% vs.17.3%, p = 0.023) and multiple organ involvement (p = 0.019), and had lower CRP levels (10.8 [0.9–27.7] mg/L vs. 1.6 [0.5–5.7] mg/L, p = 0.002) (**Table 2**). As per the definition of each group, serum IgG4 concentrations were significantly higher in the elevated serum IgG4 concentration group (122.5 [73.1–150.8] mg/dL vs. 668.0 [351.0–1362.5] mg/dL, p < 0.001). The serum IgG concentration (1243.0 [1106.3–1541.5] mg/dL vs. 1667.0 [1367.0–2033.4] mg/dL, p < 0.001) and serum IgG4/IgG ratio (0.09 [0.04–0.12] vs. 0.45 [0.23–0.72], p < 0.001) were also higher in the elevated serum IgG4 concentration group. With regard to disease activity, IgG4-RD RI was higher in the elevated serum IgG4 concentration group (4.0 [4.0–6.0] vs. 6.0 [4.0–8.0], p = 0.012).

**Table 2:** Comparison between patients with normal and elevated serum IgG4 concentration.

	Normal serum IgG4 concentration (N = 38)	Elevated serum IgG4 concentration (N = 110)	P-value
Age, years, mean ( ± SD)	52.5 ( ± 13.4)	58.9 ( ± 10.3)	0.009
Male sex, n (%)	22 (57.9)	79 (71.8)	0.112
Organ involvement, n (%)			
Lymph nodes	10 (26.3)	44 (40.0)	0.131
Orbits and lacrimal glands	8 (21.1)	39 (35.5)	0.100
Salivary glands	3 (7.9)	40 (36.4)	0.001
Kidney	5 (13.2)	19 (17.3)	0.553
Retroperitoneal fibrosis	7 (18.4)	16 (14.5)	0.570
Pancreas	1 (2.6)	19 (17.3)	0.023
Lung	5 (13.2)	15 (13.6)	0.941
Aorta	3 (7.9)	9 (8.2)	> 0.999
Bile duct	0 (0.0)	8 (7.3)	0.114
Prostate	2 (5.3)	4 (3.6)	0.647
Sinusitis	0 (0.0)	6 (5.5)	0.339
Pituitary gland	2 (5.3)	2 (1.8)	0.272
Others*	10 (26.3)	15 (13.6)	0.072
Number of organs involved			
1 organ involved, n (%)	25 (65.8)	46 (41.8)	0.019
2 organs involved, n (%)	8 (21.1)	26 (23.6)	
≥3 organs involved, n (%)	5 (13.2)	38 (34.5)	
ESR, mm/h, median (IQR)	37.5 (13.8–77.0)	32.0 (15.5–57.0)	0.868
CRP, mg/L, median (IQR)	10.8 (0.9–27.7)	1.6 (0.5–5.7)	0.002
C3, mg/dL, mean ( ± SD)	112.6 ( ± 23.5)	99.0 ( ± 33.7)	0.059
C4, mg/dL, mean ( ± SD)	27.2 ( ± 8.1)	22.4 ( ± 11.6)	0.053
IgG4 concentration, mg/dL, median (IQR)	122.5 (73.1–150.8)	668.0 (351.0–1362.5)	< 0.001
IgG concentration, mg/dL, median (IQR)	1243.0 (1106.3–1541.5)	1667.0 (1367.0–2033.4)	< 0.001
IgG4/IgG ratio, median (IQR)	0.09 (0.04–0.12)	0.45 (0.23–0.72)	< 0.001
IgG4-RD RI, median (IQR)	4.0 (4.0–6.0)	6.0 (4.0–8.0)	0.012

ESR, erythrocyte sedimentation rate; CRP, C-reactive protein; IgG4-RD, IgG4-related disease; RI, responder index.

*Detailed comparisons of other organ involvement are as follows: pleura (2.6% vs. 2.7%, p > 0.999), heart (2.6% vs. 1.8%, p > 0.999), mesentery (2.6% vs. 1.8%, p > 0.999), paraspinal mass (0.0% vs. 2.7%, p = 0.570), pericardium (2.6% vs. 1.8%, p > 0.999), testicle (5.3% vs. 0.9%, p = 0.162), gallbladder (0.0% vs. 1.8%, p > 0.999), pharynx (5.3% vs. 0.0%, p = 0.065), adnexa (2.6% vs. 0.0%, p = 0.257), bladder (2.6% vs. 0.0%, p = 0.257), colon (0.0% vs. 0.9%, p > 0.999), liver (0.0% vs. 0.9%, p > 0.999), nasal cavity (2.6% vs. 0.0%, p = 0.257), peritoneum (2.6% vs. 0.0%, p = 0.257), skin (0.0% vs. 0.9%, p > 0.999), and stomach (0.0% vs. 0.9%, p > 0.999).

### Correlation between IgG4-RD RI and serologic parameters

**Figure 1:**
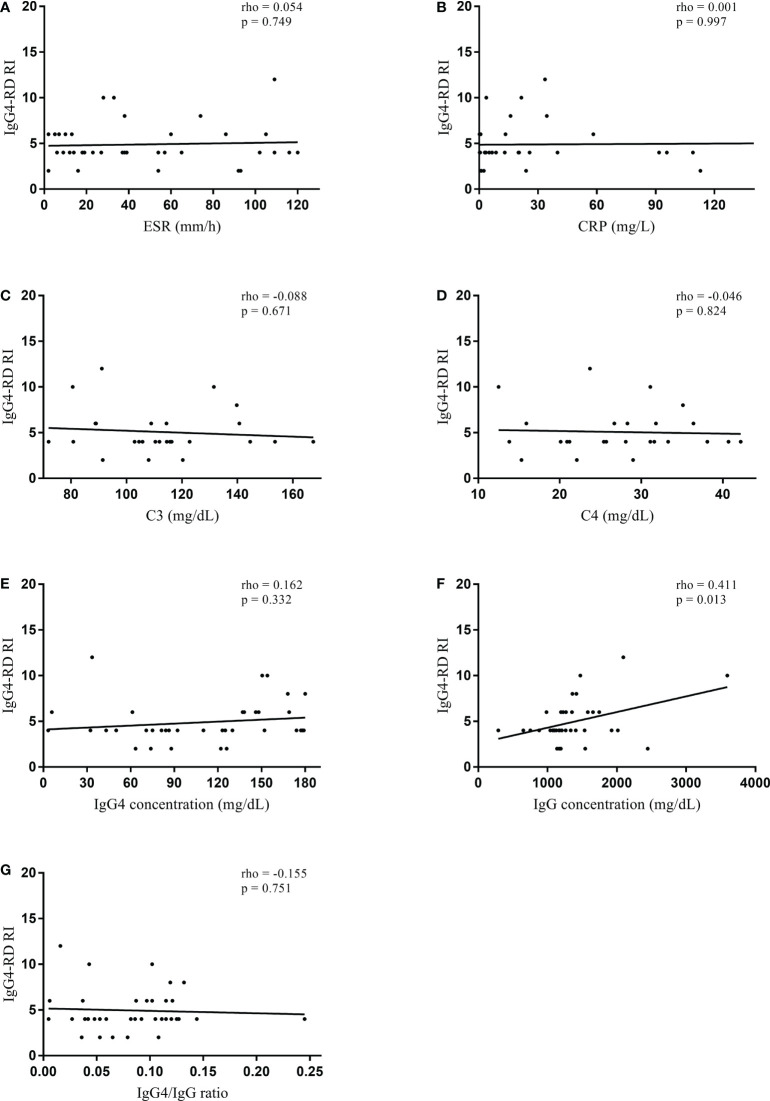
Spearman’s correlation coefficient between serologic parameters and IgG4-RD RI in the normal serum IgG4 concentration group. **(A)** ESR, **(B)** CRP, **(C)** C3, **(D)** C4, **(E)** IgG4 concentration, **(F)** IgG concentration, and **(G)** IgG4/IgG ratio. IgG4-RD, IgG4-related disease; RI, responder index; ESR, erythrocyte sedimentation rate; CRP, C-reactive protein.

**Figure 2:**
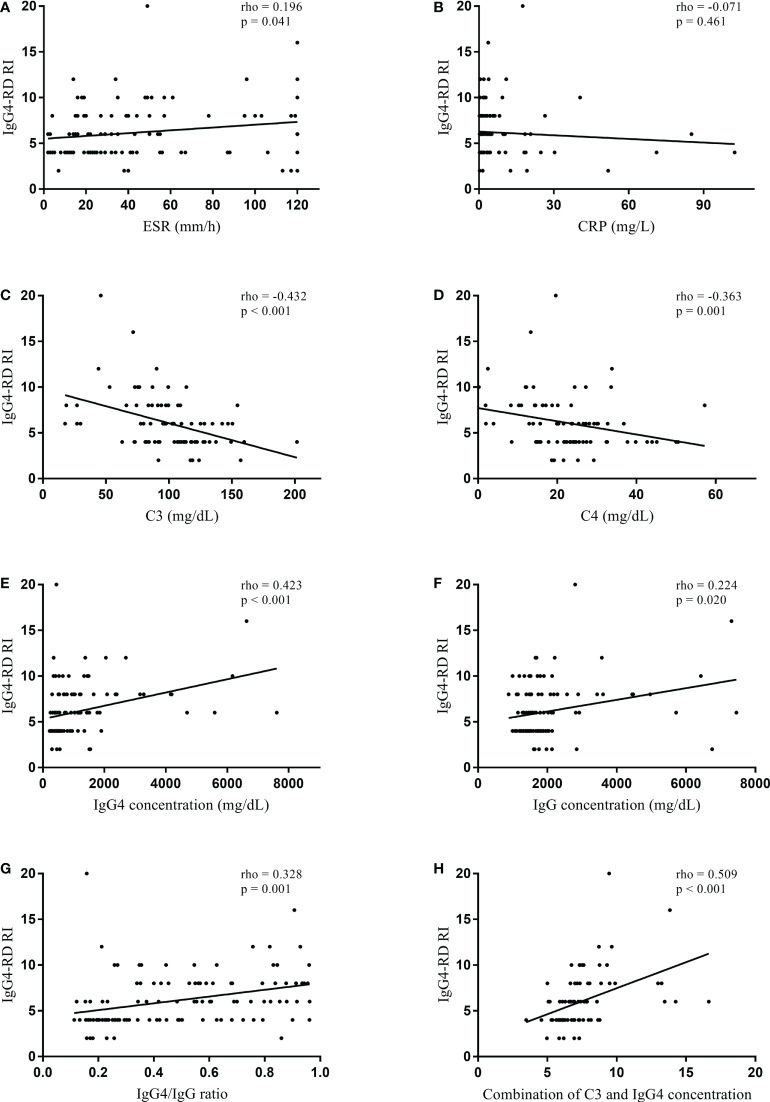
Spearman’s correlation coefficient between serologic parameters and IgG4-RD RI in the elevated serum IgG4 concentration group. **(A)** ESR, **(B)** CRP, **(C)** C3, **(D)** C4, **(E)** IgG4 concentration, **(F)** IgG concentration, **(G)** IgG4/IgG ratio, and **(H)** combination of C3 and IgG4 concentration (-0.034*C3 [mg/dL] + 0.001*IgG4 concentration [mg/dL]). IgG4-RD, IgG4-related disease; RI, responder index; ESR, erythrocyte sedimentation rate; CRP, C-reactive protein.

In the normal serum IgG4 concentration group, serum IgG4 concentration did not significantly correlate with IgG4-RD RI (rho = 0.162, p = 0.332) (**Figure 1**). However, serum IgG concentration significantly correlated with IgG4-RD RI (rho = 0.411, p = 0.013). When the correlations were tested in the patients with elevated serum IgG4 concentrations, serum IgG4 concentration significantly correlated with IgG4-RD RI (rho = 0.423, p < 0.001). Other serologic parameters including ESR (rho = 0.196, p = 0.041), C3 (rho = -0.432, p < 0.001), C4 (rho = -0.363, p = 0.001), IgG concentration (rho = 0.224, p = 0.020), and IgG4/IgG ratio (rho = 0.328, p = 0.001) also had a significant correlation with IgG4-RD RI (**Figure 2**). The combination of C3 and serum IgG4 concentration (-0.034*C3 [mg/dL] + 0.001*IgG4 concentration [mg/dL]) was selected as a composite parameter in the multivariable linear regression analysis, which had the strongest correlation with IgG4-RD RI (rho = 0.509, p < 0.001) (**Figure 2H**).

## Discussion

In this study, we found that serologic parameters that correlate with the disease activity of IgG4-RD differ between patients with normal and elevated serum IgG4 concentrations. IgG concentration was the only serologic parameter that correlated with IgG4-RD RI in patients with normal serum IgG4 concentrations. On the other hand, several serologic parameters, including ESR, C3, C4, IgG4 concentration, IgG concentration, and IgG4/IgG ratio, correlated with IgG4-RD RI in patients with elevated serum IgG4 concentrations. This finding is meaningful because it provides better insights into how serologic parameters could be interpreted in regard to disease activity in patients with IgG4-RD who have normal and elevated serum IgG4 concentrations, respectively.

In our study population, 25.7% of the patients had normal serum IgG4 concentrations. Similarly, in previous studies, normal serum IgG4 concentrations have been reported in 9.7–48.5% of patients with IgG4-RD (4–6). Thus, it is important to be aware that a substantial fraction of patients with IgG4-RD have normal serum IgG4 concentrations. There were some significant differences in characteristics between patients with normal and elevated serum IgG4 concentrations. Patients with elevated serum IgG4 concentrations were older and had a greater prevalence of multiple organ involvement than those with normal serum IgG4 concentrations. The salivary glands and pancreas were more commonly involved in patients with elevated serum IgG4 concentrations than in those with normal serum IgG4 concentrations. This is consistent with a previous cohort study, in which most of the patients (76%) were non-Hispanic whites: patients with elevated serum IgG4 concentrations were older, the proportion of patients with multiple organ involvement was higher, and the pancreas was more commonly involved than in those with normal serum IgG4 concentrations (6). Our study adds to the previous knowledge that these characteristic differences between patients with normal and elevated serum IgG4 concentrations also apply to the Asian population.

Another significant difference between patients with normal and elevated serum IgG4 concentrations was the lower CRP level in patients with elevated serum IgG4 concentrations. A recent study has shown that patients with higher number of superficial organ involvement (i.e., orbits, lacrimal glands, salivary glands, sinus and skin) have significantly lower CRP levels than those with internal organ-dominant (i.e., all other organs except the lymph nodes) involvement (14). In our study, compared with patients with normal serum IgG4 concentrations, those with elevated serum IgG4 concentrations had higher number of superficial organ involvement (0 [0–1] vs. 1 [0–1], p = 0.001) (data not shown in the Results). The difference in organ involvement pattern between the two groups could be a possible explanation for the lower CRP level in patients with elevated serum IgG4 concentrations.

Our data revealed that serologic parameters that correlate with the disease activity of IgG4-RD differ between patients with normal and elevated serum IgG4 concentrations. In patients with normal serum IgG4 concentrations, IgG concentration (rho = 0.411, p = 0.013), but not IgG4 concentration (rho = 0.162, p = 0.332), significantly correlated with IgG4-RD RI. In contrast, in patients with elevated serum IgG4 concentrations, multiple serologic parameters including IgG4 concentration (rho = 0.423, p < 0.001) correlated with IgG4-RD RI. The precise role of IgG4 on the disease pathogenesis of IgG4-RD remains unclear; an adoptive transfer model has shown that injection of patient IgG4 in neonatal BALB/c mice results in pancreatic and salivary gland injuries, suggesting a pathogenic role of IgG4 (15); while an anti-inflammatory role of IgG4 has also been suggested (16). Further studies are needed to determine whether the serum IgG4 plays a different role in patients with normal and elevated serum IgG4 concentrations. Regardless, our study shows that serum IgG4 concentration correlates with disease activity only in patients with elevated serum IgG4 concentrations.

Similar to the correlations observed in patients with elevated serum IgG4 concentrations in our study, a previous study consisting of 72 patients with IgG4-RD (68 [94.4%] patients with elevated serum IgG4 concentrations) showed that serum IgG4 concentration, serum IgG concentration, C3, and C4 correlate with IgG4-RD RI (17). We further advanced this finding by investigating whether the combination of the serologic parameters could yield a stronger correlation with disease activity and found that a combination of C3 and IgG4 concentration (rho = 0.509) had a stronger correlation with IgG4-RD RI than C3 alone (rho = -0.432) or IgG4 concentration alone (rho = 0.423). A previous study on 8 patients with IgG4-RD suggested that serum IgG4 could be associated with complement activation (18). Subsequent study on 12 patients with IgG4-RD from the same group reported that serum IgG4, particularly that with flucosylation change, may be associated with complement activation (19). In contrast, more recent studies with larger number of patients (n = 85 (20); and n = 328 (21)) suggested that hypocomplementemia in IgG4-RD is associated with IgG subclasses other than IgG4. Indeed, immune complexes that contain IgG4 bind complement weakly; hypocomplementemia in IgG4-RD results from immune complexes containing IgG1 or IgG3 rather than IgG4 (2, 22, 23). The mechanistically independent action of C3 and serum IgG4 could explain why the combination of these two parameters showed a stronger correlation with disease activity.

We used a cut-off of 201 mg/dL to categorize patients into normal serum IgG4 concentration and elevated serum IgG4 concentration groups. Diagnostic criteria for IgG4-RD, established a decade ago, used a value of 135 mg/dL for the cut-off of elevated serum IgG4 concentration (24). One might argue that this cut-off value should be used for categorizing patients with normal and elevated serum IgG4 concentrations; however, this cut-off value was calculated in a study in which serum IgG4 concentrations were measured using a Binding Site assay (Binding Site, Birmingham, UK) (9). More recent studies have shown that the reference range of serum IgG4 concentration varies according to the measurement method; serum IgG4 concentrations are significantly higher when measured with the Siemens assay than with the Binding Site assay (25, 26). In line with these observations, the 2019 ACR/EULAR classification criteria for IgG4-RD did not state 135 mg/dL as the cut-off for the upper normal limit of serum IgG4 concentration but adopted the reference range of each assay as the cut-off for classification (12). As serum IgG4 concentrations were measured using Siemens assay in our study, we used the concentration of 201 mg/dL as the cut-off for the upper normal limit (25).

Our study had some limitations. First, the possibility of prozone phenomenon in the patients with normal serum IgG4 concentrations cannot be excluded (27). As this was a retrospective study using data obtained at the time of diagnosis of IgG4-RD, we are uncertain whether diluting method was appropriately performed to avoid the prozone phenomenon. Studies have shown that elevated serum IgG4 concentrations decrease during glucocorticoid treatment in all patients with IgG4-RD (28, 29). Therefore, if the normal serum IgG4 concentrations were the result of prozone phenomenon, the concentrations during the glucocorticoid treatment would increase initially and decrease subsequently. However, in the patients with normal serum IgG4 concentrations in our study, the increase in serum IgG4 concentrations during the early phase of glucocorticoid treatment was not observed in any of the patients (data not shown in the Results). This observation can be a clue to infer that the normal serum IgG4 concentrations were not likely the result of prozone phenomenon. Second, only the traditional serologic biomarkers were available, and we lacked several types of data, such as interleukin-6, soluble interleukin-2 receptor and CC-chemokine ligand 18, which have been more recently suggested as putative biomarkers of disease activity (8, 17, 30–32). However, considering that the recently suggested putative biomarkers are not as easily available as the traditional serologic biomarkers in routine clinical practice, our data are still clinically relevant for routine clinical practice. Third, although we found that serum IgG concentration, but not serum IgG4 concentration, correlated with disease activity in patients with normal serum IgG4 concentrations, an explanation for this correlation could not be drawn from our data. Further studies evaluating the mechanisms underlying this correlation would be helpful.

In conclusion, we demonstrated that serologic parameters correlate differently with disease activity depending on serum IgG4 concentrations. IgG concentration was the only serologic parameter that correlated with IgG4-RD RI in patients with normal serum IgG4 concentrations, while multiple serologic variables including ESR, C3, C4, IgG4 concentration, IgG concentration, and IgG4/IgG ratio correlated with IgG4-RD RI in patients with elevated serum IgG4 concentrations. In the latter group, a combination of C3 and IgG4 concentration as a composite parameter showed the strongest correlation with IgG4-RD RI. These findings could be useful for a more accurate serologic assessment of disease activity in patients with normal and elevated serum IgG4 concentrations, respectively.

## Data availability statement

The original contributions presented in the study are included in the article/Supplementary Material. Further inquiries can be directed to the corresponding authors.

## Ethics statement

The studies involving human participants were reviewed and approved by the Institutional Review Board (IRB) of Gangnam Severance Hospital (IRB No: 3-2022-0247). Written informed consent for participation was not required for this study in accordance with the national legislation and the institutional requirements.

## Author contributions

OCK, M-CP and Y-GK contributed to the conception and design of the study. OCK, M-CP and Y-GK participated in acquisition of data, data analyses and data interpretation. OCK, M-CP and Y-GK wrote the manuscript. All authors contributed to the article and approved the submitted version.

## Funding

Y-GK received grants from the National Research Foundation of Korea (NRF-2021M3A9G1026605).

## Conflict of interest

The authors declare that the research was conducted in the absence of any commercial or financial relationships that could be construed as a potential conflict of interest.

## Publisher’s note

All claims expressed in this article are solely those of the authors and do not necessarily represent those of their affiliated organizations, or those of the publisher, the editors and the reviewers. Any product that may be evaluated in this article, or claim that may be made by its manufacturer, is not guaranteed or endorsed by the publisher.
